# A novel transformer model of protein domains for viral taxonomy classification

**DOI:** 10.1093/bioinformatics/btag291

**Published:** 2026-07-07

**Authors:** Jihye Shin, Qingyang Xiao, Yuzhen Ye

**Affiliations:** Luddy School of Informatics, Computing and Engineering, Indiana University, Bloomington, IN 47408, United States; Luddy School of Informatics, Computing and Engineering, Indiana University, Bloomington, IN 47408, United States; Luddy School of Informatics, Computing and Engineering, Indiana University, Bloomington, IN 47408, United States

## Abstract

**Motivation:**

Viruses with carefully curated taxonomic assignments (such as those in the ICTV taxonomy) still represent only a small fraction of viruses identified through sequencing data from virome or microbiome projects. It is therefore critical to develop methods that can assign viruses at multiple taxonomic ranks, so that a virus deemed novel at a given rank may still be placed into a higher-level taxon. Sequence-similarity–based approaches can classify viruses that share substantial genomic similarity with known viruses (e.g. those belonging to the same species or genus); however, their performance drops significantly when applied to more divergent viruses. Recent deep learning models, such as ViTax, which utilize DNA language models, aim to address these limitations, but their performance also degrades when applied to novel viruses lacking genus-level similarity to known references. Proteins are more conserved than genomic sequences, and the multiple proteins encoded by a virus can be leveraged to reveal evolutionary relationships among viruses.

**Results:**

We propose a new tool, D2T (Domain-to-Taxonomy), that leverages recent advances in protein language models to improve viral taxonomic assignment. D2T represents a virus as a sequence of protein domain tokens and learns a transformer-based model for taxonomic classification. Experiments on multiple closed-set and open-set datasets show that D2T excels at assigning higher-level taxonomic labels (family and above). Furthermore, by combining D2T with Kraken2, which performs well at the genus level, the hybrid method (K+D2T) achieves accurate viral taxonomic classification across multiple taxonomic ranks.

**Availability and Implementation:**

D2T is available as a GitHub repository at https://github.com/mgtools/D2T.

## 1 Introduction

Viruses are the most abundant and diverse biological entities on Earth, infecting all forms of life and playing essential roles in evolution, ecology, and health. The virome (Wommack et al. 2012), the complete collection of viruses in a given environment, such as the human body, oceans, or soil, includes both viruses that infect host cells and phages that shape microbial communities. Enabled by metagenomic sequencing and advanced computational analysis, virome projects (Delwart 2013) seek to systematically characterize viral diversity, uncover virus–host interactions, and understand how viruses influence ecosystems and disease processes. The human virome is considered to be an integral part of human health and disease (Gholamzad et al. 2024). The human virome is responsible for regulating the growth and responses of the host’s immune system, and plays a crucial role in the development of diseases, including inflammatory bowel disease (IBD) and Crohn’s disease.

Taxonomy is essential for understanding virus classification and evolution (Gorbalenya et al. 2020). Taxonomy is the hierarchical classification of organisms based on shared characteristics, often following a predefined naming system. The International Committee on Taxonomy of Viruses (ICTV) (Simmonds et al. 2023) creates the official hierarchical taxonomy (including realm, kingdom, phylum, class, order, family, genus and species) for viruses, based on viral morphology, genome type, replication strategy, and evolutionary relationships. It provides a standardized framework that allows for consistent naming and categorization. Despite the large number of putative viruses that have been identified from sequencing projects and deposited into the NCBI database, classification of viruses has lagged. There are other classification systems like the Baltimore classification that may not follow evolutionary relationships (Koonin et al. 2021).

Similarity-based methods for taxonomic assignments can be applied to classify viruses by searching against reference viruses. Kraken2 (Wood et al. 2019) is a k-mer–based taxonomic classifier that performs exact k-mer matching rather than alignment. It builds a minimizer index from reference genomes, storing for each minimizer the Lowest Common Ancestor (LCA) of genomes containing it. For each query sequence, Kraken2 extracts k-mers, looks up their minimizers, and aggregates the resulting LCA assignments into clade scores. When a confidence score threshold is specified, where the confidence score is the fraction of k-mer votes supporting a clade, the sequence is classified at the lowest taxonomic rank in the lineage with a confidence score meeting or exceeding the threshold. Otherwise, the sequence remains unclassified. CAT (Von Meijenfeldt et al. 2019) applies similar LCA algorithm but relies on similarity between ORFs (open reading frames) translated from genomic sequences. The accuracy of these similarity-based methods relies on the completeness and accuracy of the reference database of viruses with taxonomy information.

Learning-based methods on the other hand may leverage machine-learning models to classify viruses. PhaGenus (Guan et al. 2023) is a transformer model that uses protein clusters as features for classification of phages (bacterial viruses) at genus rank. Another recently developed learning-based method ViTax (He et al. 2025) was built based on a genomic foundation model (HyenaDNA (Nguyen et al. 2023)) and supervised prototypical contrastive learning to learn an embedding space. During inference, ViTax performs adaptive hierarchical assignment using an LCA-based taxonomy belief mapping tree: it first attempts genus-level prediction and, when the genus-level confidence is insufficient, backs off to higher taxonomic ranks until a reliable assignment is obtained. ViTax (He et al. 2025) was reported to outperform other existing methods including Kraken2, CAT and PhaGenus. However, the comparison was done only on double-stranded DNA viruses.

There are several challenges associated with the taxonomic assignment of viruses. Viral taxonomy is hierarchical, and taxonomic ranks reflect degrees of evolutionary relatedness among viruses. However, viruses mutate rapidly, and related viruses can quickly lose sequence similarity, particularly at the nucleotide level. Protein sequence similarity can be used to infer more distant relationships, such as those at the genus level. Nevertheless, inferring deeper evolutionary relationships beyond the genus level remains challenging due to the lack of genes conserved across all viral genomes (Simmonds et al. 2023). For example, genes involved in virion morphogenesis are informative for studying DNA viruses, whereas genes encoding genome replication machinery are more useful for RNA viruses. Moreover, even hallmark genes and their encoded proteins may become too divergent to be reliably identified and used for taxonomic assignments at higher taxonomic ranks.

Recently developed protein language models (PLMs) have enabled a wide range of applications through the protein contextual embeddings they generate. Commonly used PLMs include ProtTrans (Elnaggar et al. 2022), which was trained to produce informative embeddings for downstream tasks such as secondary structure and subcellular localization prediction, and ESM-2 (Lin et al. 2023), which was trained to generate embeddings that facilitate protein structure prediction from sequence alone. We previously developed DCTdomain, which leverages the contextual embeddings produced by ESM-2; however, rather than using whole-protein representations, it first segments proteins into domains (based on predicted contact maps from ESM-2) and then applies discrete cosine transformation (DCT) to the vectorized residue embeddings within each domain to infer domain-level contextual vectors (*domain fingerprints*) (Iovino et al. 2024). We previously showed that domain fingerprints can enable sensitive homology searches (Iovino et al. 2024). Here, we develop D2T, a model that uses protein domain embeddings as input and learns complex patterns reflecting viral genome modularity for taxonomic assignment. Benchmarking D2T against existing methods demonstrates that contextual information from protein domains can be leveraged to achieve accurate taxonomic assignments, particularly at higher taxonomic ranks (beyond genus), where protein-level sequence similarity is low.

## 2 Methods

### 2.1 Overview of the model

**Figure 1:**
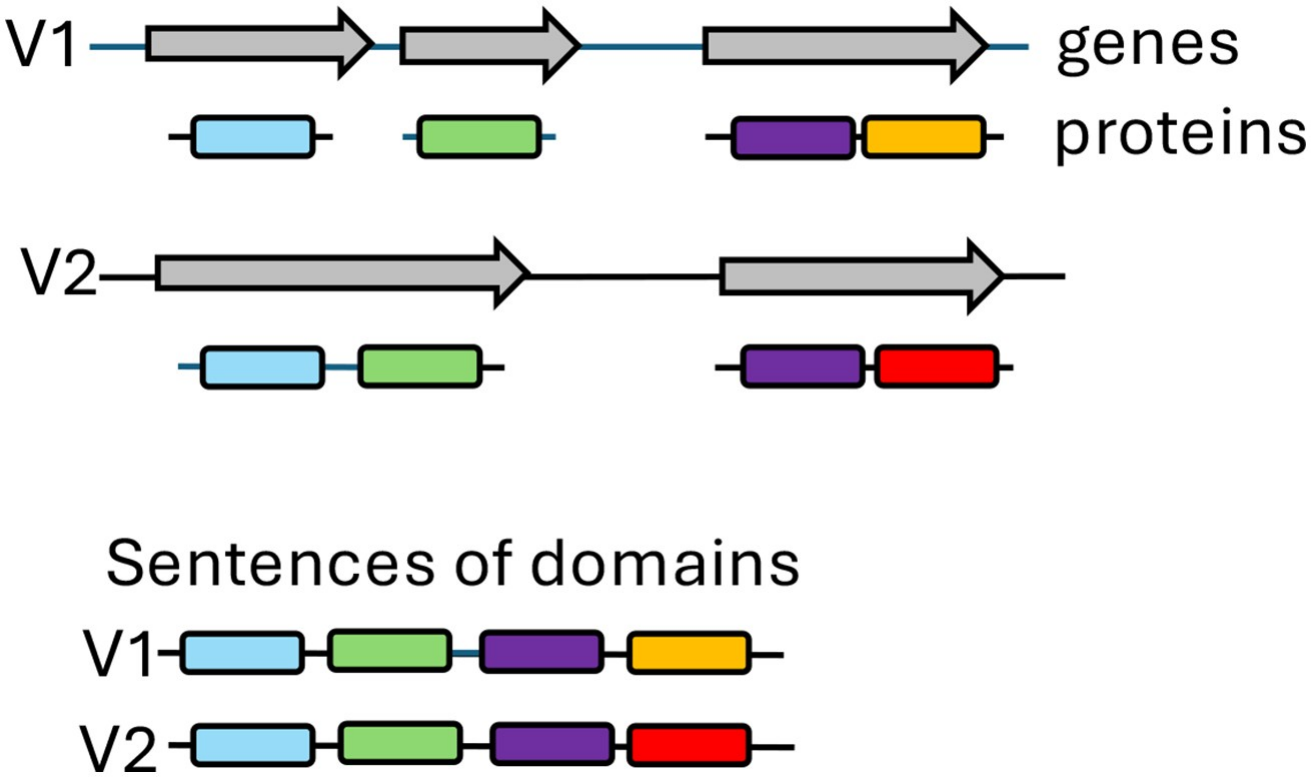
Using domain fingerprints for viral taxonomic classification. Our transformer-based AI model D2T uses domain tokens. In this plot, different protein domains are shown as rectangles in different colors. Gray arrows represent genes. V1 and V2 are toy viral genomes.

**Figure 2:**
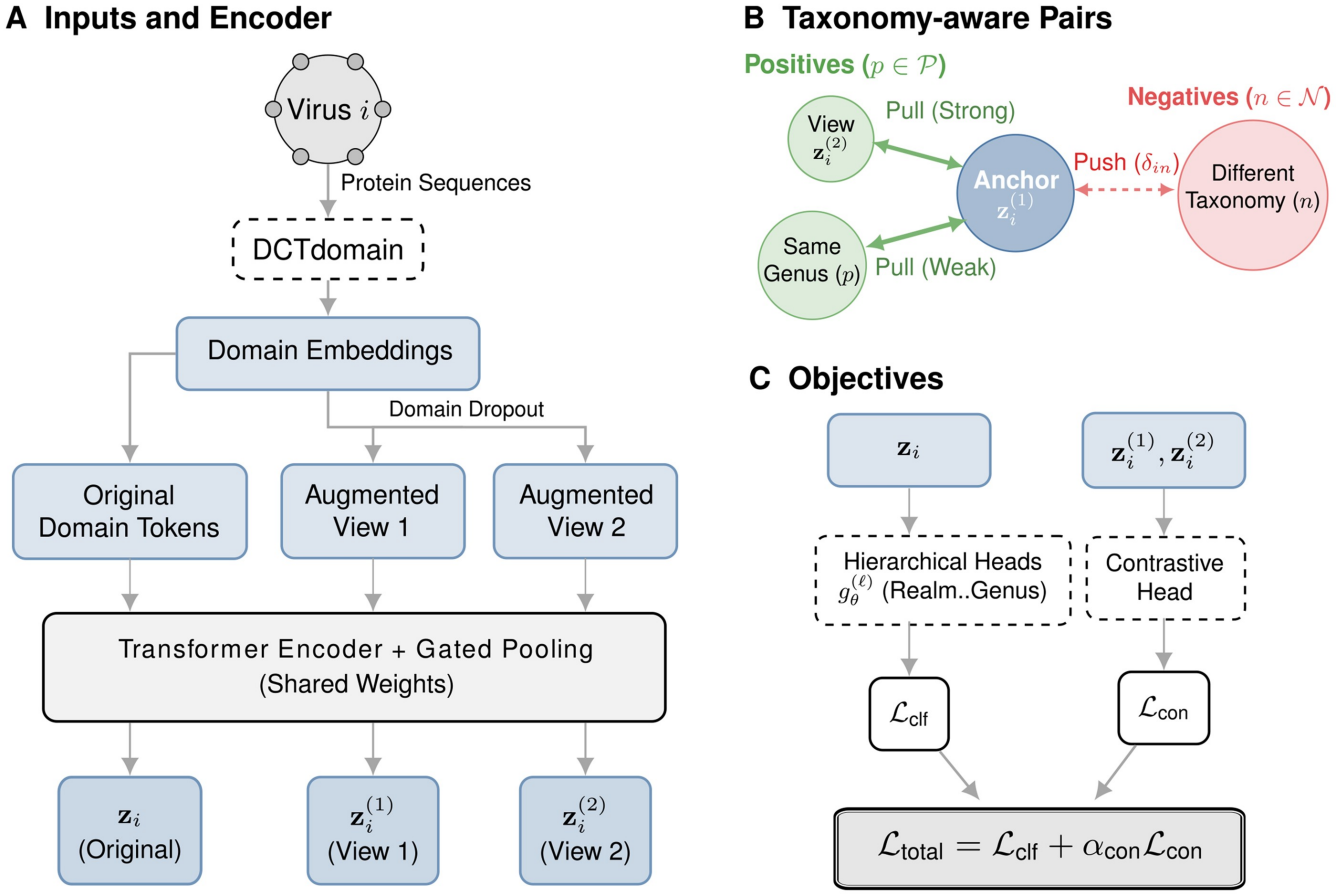
Overview of the D2T framework for viral taxonomy classification. D2T is based on a transformer model of protein domain tokens for taxonomic assignment of viruses. Strategies including using contrastive learning and hierarchy-aware loss functions are used for efficient learning of the latent embedding for classification. (A) Viral protein sequences are tokenized into domains. The model utilizes sentences of domains as the input. The model applies domain dropout to generate augmented views, which are processed alongside original tokens by a shared Transformer encoder and gated attention pooling to produce the final virus embeddings. (B) The contrastive module pulls the anchor embedding towards its augmented view (strong positive) and samples from the same genus (weak positive), while pushing away negatives from different taxonomy. The taxonomic distance ($\delta_{ij}$) determines how strongly they are pushed apart. (C) The model is optimized using a joint objective function combining hierarchical classification ($L_{\text{clf}}$) and contrastive learning ($L_{\text{con}}$) to learn latent representations.

Our method, D2T, represents a virus as a sentence of protein domains (see Fig. 1 for an illustration). D2T employs a transformer-based deep learning model enhanced with contrastive learning (see Fig. 2). We further propose a hybrid approach that leverages the complementary strengths of D2T and a similarity-search–based method (Kraken2) to achieve accurate classification across all taxonomic ranks. This hybrid approach is referred to as K+D2T.

### 2.2 Representing viral genomes as sentences of domain tokens

For each viral genome, all of the proteins it encodes are represented as domain fingerprints which are then used as the inputs to D2T for viral taxonomic assignments. Specifically, we used DCTdomain (Iovino et al. 2024) to derive domains for all the proteins encoded by each viral genome, and encode each domain as a fingerprint. This way, a domain of any length is represented as a vector of fixed dimension (e.g. 480). A practical advantage of using protein domains as tokens is that each virus now can be represented as a relatively short sequence of such tokens (unlike using each residue as a token). Such representation of proteins could be important for training a model when the training sample size, here the number of viruses with taxonomic labels, is limited.

### 2.3 Transformer encoder and pooling

For each virus *i*, we construct a padded domain-token sequence $X_{i}$ and a padding mask indicating non-informative positions. We encode $X_{i}$ using a lightweight Transformer (Vaswani et al. 2017) with 2 layers, 4 attention heads, and hidden size $d_{\text{model}}=480$. Rotary positional embeddings (Su et al. 2024) are applied inside self-attention to handle variable-length sequences without explicit absolute positional encodings. Each layer includes multi-head self-attention, a feed-forward block of size $2\times d_{\text{model}}$, residual connections, layer normalization, and dropout 0.1. The token-level outputs are aggregated into a single virus embedding $z_{i}$ using gated attention pooling (Ilse et al. 2018), where each token receives a learned weight (tanh path gated by a sigmoid path) and the weighted sum becomes $z_{i}$. Padding tokens are masked out during pooling.

### 2.4 Supervised learning with taxonomy-aware contrastive learning

There are some challenges of viral taxonomy, which are the hierarchical structure, limited labels with missing values, and class imbalance. To address these, we propose a taxonomy-aware weighted margin NT-Xent loss. Unlike the standard NT-Xent (Chen et al. 2020), which treats all negative pairs equally, our objective incorporates taxonomic distances to weight negative samples and enforce distance-dependent margins.

For contrastive learning, we generate two stochastic views by applying domain-token dropout before the Transformer encoder. Each view is passed through the same Transformer and pooling (shared weights) to obtain two embeddings, which form a strong positive pair. Each embedding becomes an anchor in turn. For each anchor, its paired view is the strong positive, same-genus samples are weak positives, and the rest are negatives.

The classification loss uses the embedding from the original (non-dropout) input, while the contrastive loss uses the two view embeddings. Embeddings are $\ell_{2}$-normalized before computing similarities.

#### 2.4.1 Taxonomic distance with missing labels

We define a taxonomic distance metric that is robust to missing annotations. For any pair of samples *i* and *j* with hierarchical labels $y_{i}$ and $y_{j}$ across *L* levels, we determine the depth of the longest contiguous taxonomic ranks (starting from Realm) where both labels are observed. Let $P_{ij}$ be this depth of defined (non-missing) ranks for both viruses, and $C_{ij}$ be the depth of their LCA. The normalized taxonomic distance is defined as:


(1) $$\delta_{ij}=1-\frac{C_{ij}}{P_{ij}},\;\text{where}\;\delta_{ij}\in [0,1].$$

Pairs with insufficient information ($P_{ij}=0$) are excluded from the negative set. Genus-match positives are still applied as positive pairs when genus labels are available, even when $P_{ij}=0$.

#### 2.4.2 Taxonomy-aware contrastive loss formulation

We extend the contrastive framework by modifying the positive and negative definitions based on $\delta_{ij}$. Let $z_{i}$ be the $\ell_{2}$-normalized embedding of sample *i* and $S_{ij}=z_{i}\cdot z_{j}$ be the cosine similarity. Here, each anchor *i* denotes one embedding (one view) in the batch. Positives and negatives are defined relative to this anchor. The modified loss for an anchor *i* is formulated as:


(2) $$L_{\text{con},i}=-\text{log}\;\frac{\sum_{p\in P(i)}\omega_{ip}^{+}\;\text{exp}(s_{ip}/\tau )}{\sum_{p\in P(i)}\omega_{ip}^{+}\;\text{exp}(s_{ip}/\tau )+\sum_{n\in N(i)}\omega_{in}^{-}\;\text{exp}((s_{in}+m_{in})/\tau )}$$

In practice, we use logsumexp aggregation for the numerator and denominator to ensure numerical stability. The loss is then averaged over all anchors in the mini-batch. Here, τ is the temperature parameter, and the components are defined as follows:

- Positives (P): We generate two stochastic views per sample via domain dropout. The paired views are treated as positives. In addition to standard augmentation views, we include samples as positives sharing the same genus when genus labels are available. These are weighted by $\omega_{ip}^{+}$ (augmented-pair weight 1.0, genus-match weight 0.5).
- Negatives (N): The negative set consists of taxonomically distinct pairs ($\delta_{in}>0$), where *n* denotes an index in the negative set N(i) for anchor *i*. We weight these pairs by their taxonomic distance using $\omega_{in}^{-}=\text{exp}(\lambda \cdot \delta_{in})$, where λ is a scalar hyperparameter to prioritize distant negatives.
- Distance-dependent margin ($m_{in}$): We apply a margin $m_{in}$ to adjust the similarity constraint based on taxonomic distance between anchor *i* and negative *n*. The margin is determined by round-binning $\delta_{in}$ into K=L+1 bins and mapping them to a linear schedule. The margin is added to the negative similarity, acting as a penalty that forces the model to learn stricter separation for taxonomically distant pairs, so larger biological distance yields stronger separation in the embedding space. In this way, the similarity score of distant negatives is artificially inflated, compelling the model to push them further apart to minimize the loss.

#### 2.4.3 Total objective

The model is trained to simultaneously optimize representation learning and taxonomic prediction. The virus embedding $z_{i}$ is fed to a hierarchical classification head $g_{\theta }^{\left(\ell \right)}$ at each taxonomic level ℓ. The classification loss is a weighted average of cross-entropy losses across levels, ignoring missing labels (encoded as ⊥). We configure $w_{\ell }$ to assign higher weights to higher taxonomic ranks to ensure structural consistency (predict higher level more accurately), while assigning a high weight to the Genus level to maintain fine-grained prediction accuracy in the lowest rank. We use fixed default rank weights $w_{\ell }=\{0.18,0.17,0.15,0.13,0.11,0.10,0.16\}$ (realm to genus), normalized over levels with valid labels. These rank weights were determined empirically to balance between maintaining high-level accuracy and preserving sufficient gradient signals for finer, more challenging ranks. See the performance comparison in Fig. S1.

Let $B_{\ell }=\{i|y_{i}^{\left(\ell \right)}\neq ⊥\}$ be the set of samples with observed labels at level ℓ. To handle cases where a mini-batch may contain no samples for a specific level, we define the set of valid levels as $L_{\mathrm{valid}}=\{\ell ||B_{\ell }|>0\}$. The classification loss is defined as a weighted average over levels:


(3) $$L_{\text{clf}}=\frac{1}{\sum_{\ell \in L_{\mathrm{valid}}}w_{\ell }}\sum_{\ell \in L_{\mathrm{valid}}}w_{\ell }\cdot \frac{1}{\left|B_{\ell }\right|}\sum_{i\in B_{\ell }}\text{CE}(g_{\theta }^{\left(\ell \right)}(z_{i}),y_{i}^{\left(\ell \right)}),$$

where CE denotes the Cross-Entropy loss.

The final objective combines the classification and contrastive losses:


(4) $$L_{\text{total}}=L_{\text{clf}}+\alpha_{\text{con}}L_{\text{con}}.$$

where $\alpha_{\text{con}}$ is a parameter that controls the relative contribution of the contrastive loss to the total loss.

### 2.5 Datasets for training and testing

We used taxonomic assignments from ICTV (Lefkowitz et al. 2018) as ground-truth labels for training and testing. Specifically, we leveraged multiple ICTV releases including MSL #38 (referred as MSL38 below for simplicity), MSL #39 release v4 (MSL39), and the most recent MSL #40 release v2 (MSL40) to construct *closed-set* and *open-set* datasets. In the closed-set setting, the test split contains viruses belonging to genera that are seen in the training split. In contrast, in the open-set setting, the test split contains viruses from *novel* genera, i.e. genera that don’t appear in the training split.

To provide consistent and fair comparison of our methods with ViTax (He et al. 2025) and other methods, we first used the closed-set and open-set that were previously constructed based on MSL38 (He et al. 2025). The training split, closed-set testing split and open-set testing split contain 2462, 1516, 785 samples, respectively. This dataset only contains double-stranded DNA viruses.

In addition, we constructed a new training and test dataset based on MSL39 similarly to the approach reported in He et al. (2025), to test the performance of our new methods. Importantly, this dataset includes all groups of viruses collected in ICTV, including DNA viruses (*Adnaviria*, *Duplodnaviria*, and *Varidnaviria*) and RNA viruses (*Riboviria* and *Monodnaviria*). Genera represented by exactly one isolate were first assigned to an open-set subset. The remaining data were randomly shuffled and split into training and test sets with a target ratio of 3:2. In addition, for each taxonomic rank, any test samples whose rank labels were absent from the training set were moved into the training set to ensure a closed-set evaluation. The resulting dataset consists of 8590 training samples, 5728 test samples, and 1899 open-set samples. This new dataset is important for testing if our methods can be applied to all groups of viruses, not just the double-stranded DNA viruses.

**Figure 3:**
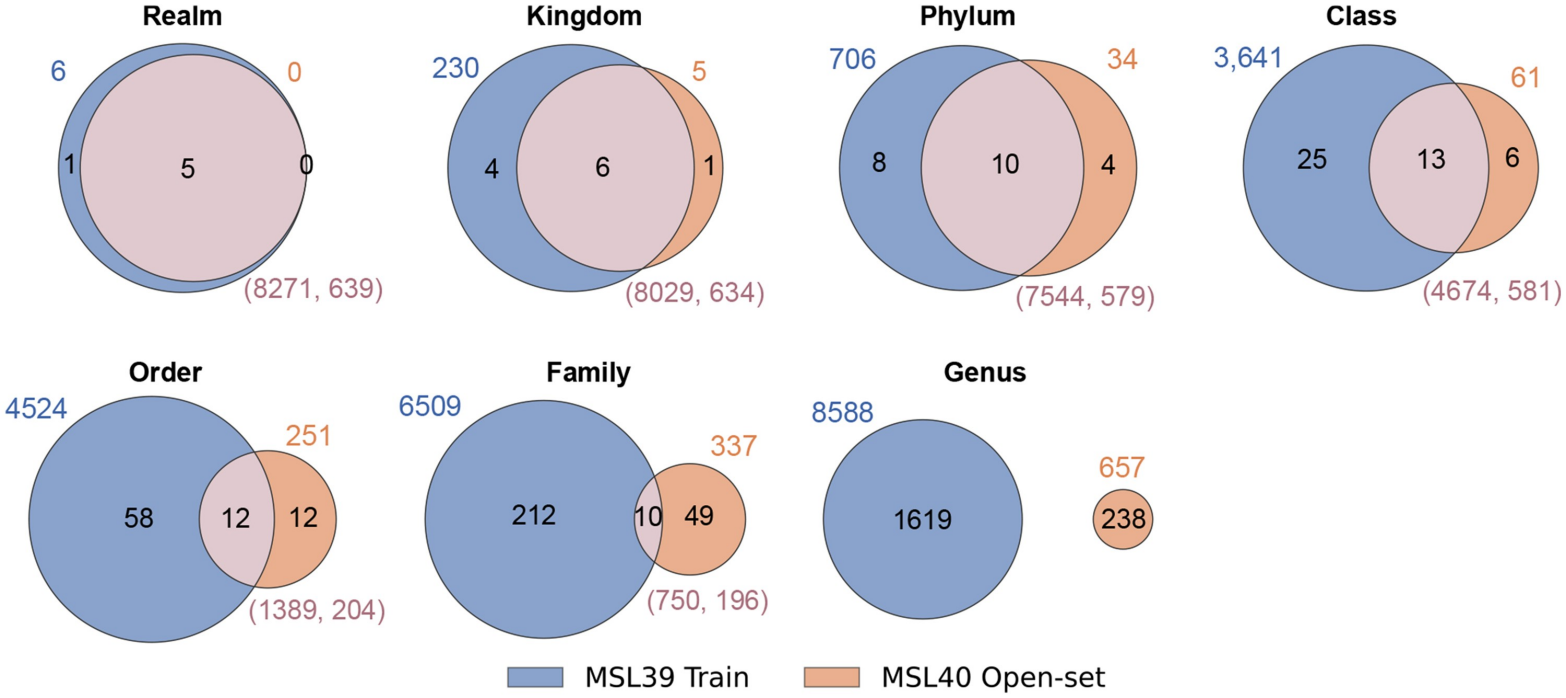
MSL39 training data vs. MSL40 open-set data with new genera. Venn diagrams comparing taxonomic coverage between the MSL39 training set and the MSL40 open-set at each taxonomic rank. Numbers inside the Venn diagrams indicate the number of unique taxa at the corresponding rank. Numbers shown outside the diagrams in the same color represent the number of virus samples associated with each region. The numbers in parentheses $\left(n_{\text{train}},n_{\text{open}}\right)$ denote the numbers of virus samples in the MSL39 training set and the MSL40 open-set respectively that share taxa in the intersection.

Finally we constructed a temporally grounded open-set setting based on the two most recent ICTV releases to mimic real-world applications. Specifically, isolates that are newly introduced in MSL40 and absent from MSL39 are treated as open-set samples and are never observed during training. Notably, all 1563 isolates in this MSL40 subset correspond to newly defined viral species in the ICTV taxonomy. To define a strict and unambiguous open-set evaluation, we focused on isolates belonging to newly introduced genera in MSL40. In total, 238 genera are newly defined in MSL40, and the 657 isolates assigned to these genera are treated as true open-set samples for evaluation in our experiments. Figure 3 shows the distribution of novel viruses across different taxonomic ranks for the open-set testing over diverse viruses.

We used all the isolates that have GenBank accessions. For each accession, we retrieved protein FASTA sequences from NCBI using the GenBank accessions provided by ICTV. For accessions without available protein FASTA, we downloaded the corresponding genome sequences and predicted protein-coding genes using prodigal-gv (v2.11.0) in metagenome mode (-p meta) (Hyatt et al. 2010, Camargo et al. 2024). This gene-calling step was used to account for virus-specific features such as alternative genetic codes and gene structures commonly observed in giant viruses. DCTdomain was then applied to generate domain fingerprints for all the proteins, and the domain fingerprints are used as inputs to our new method D2T.

### 2.6 Evaluation and comparison with existing methods

We used standard classification metrics, including accuracy, balanced accuracy, precision, recall, Macro F1, and Matthews correlation coefficient (MCC) to evaluate the accuracy of different models. As virus taxonomy annotations are often incomplete and not all viral isolates are annotated at every taxonomic rank, we excluded the missing labels in the ground truth for computing these metrics.

To faithfully report the performance of different methods in the open-set setting (reflecting real-world practice), samples from novel taxa are included and counted as incorrect, since they cannot be correctly predicted under a closed label space. In the open-set setting, the test split contains viruses from novel genera (i.e. genera unseen in the training split), which may be novel or known at higher taxonomic ranks. For example, a new virus may be novel up to the order rank (making it novel at the order, family, and genus levels) while remaining known at the realm, kingdom, phylum, and class levels.

For the open-set cases, we report an additional accuracy where only samples known at a given rank (potentially unknown at lower ranks) are included. This accuracy reflects a more optimistic scenario when the novelty information is available. Along with accuracy, we also report the proportion of novel samples in the test set to indicate problem difficulty, as models tend to perform worse on test sets containing a higher fraction of novel samples.

For comparison, we included two methods, ViTax (He et al. 2025) and Kraken2 (Wood et al. 2019) that represent learning-based and similarity-based methods for taxonomic assignments, respectively. As we were unable to use the training code provided by ViTax, we used the pre-split MSL38 dataset and the released pre-trained ViTax model provided in the ViTax GitHub repository, to enable a direct and fair comparison. So comparison with ViTax is limited to the test-sets derived from MSL38. For comparison with Kraken2, we used the viruses in the training set to create reference databases for classification of the viruses in the test splits. We compared our methods with Kraken2 on all test-sets.

### 2.7 The hybrid approach K+D2T

We observed that our protein-domain–based model, D2T, excels at assigning higher-level taxonomic labels to viral genomes that lack significant genomic similarity to known viruses, but may be confused at the genus level, where Kraken2 performs well (see Results). To best leverage the strengths of both approaches, we developed a simple hybrid method, K+D2T, which applies D2T only to sequences that remain unclassified by Kraken2.

We also evaluated other similarity-search–based tools for taxonomic assignment. Specifically, we tested whether skani (Shaw and Yu 2023), a recently developed tool for fast computation of average nucleotide identity (ANI), could be used to assign taxonomy to viruses that share substantial similarity with known genera. The results showed that directly transferring taxonomic labels based on computed ANI performed worse than Kraken2. Therefore, in this study, we use Kraken2 both for comparison and for integration with D2T.

### 2.8 Interpretability of the models

We used attention-based and SHapley Additive exPlanations (SHAP)-based attribution scores (Lundberg and Lee 2017) to provide interpretations for D2T predictions. These scores can be used to estimate the relative contribution of individual proteins to taxonomic predictions. Protein-level importance scores are computed by summing SHAP values across domains within each protein, which reflects how much that protein shifts the model prediction toward or away from a specific taxonomic label. Proteins with large positive SHAP values provide strong evidence supporting the predicted taxonomy, whereas proteins with values close to zero have limited influence on the decision. As a result, highly ranked proteins can be interpreted as candidate marker proteins that capture lineage-specific sequence or domain patterns learned by the model. We implemented it using gradient SHAP from Captum API (Kokhlikyan et al. 2009).

### 2.9 Implementation and availability

We implemented D2T in Python with PyTorch (Paszke et al. 2019) and used scikit-learn (Pedregosa et al. 2011) for K-fold splits. Domain-token sequences are padded for batching and a padding mask excludes padded positions from attention and pooling. We fixed the random seed to 42 for reproducibility. With pre-split files, we performed 5-fold cross-validation only on the training set and select the checkpoint with the lowest validation loss for final evaluation on the held-out test set.

Experiments were run on NVIDIA A40 GPUs (46GB per GPU). On this hardware, training the model takes up to 9 hours for all viruses in the MSL39 dataset, depending on the number of epochs required before early stopping. During inference, the pipeline first derives domain fingerprints from protein sequences using DCTdomain. While this step incurs some computational cost, it processes sequences at a reasonable speed (e.g. taking 65 minutes for 79 222 proteins encoded by 657 novel viruses from MSL40, or approximately 0.05 seconds per protein). Given these extracted fingerprints, D2T then provides rapid taxonomic assignment, taking about 6 seconds for the same 657 viruses.

Hyperparameters were manually tuned on MSL38 dataset using the training and validation folds only, and the selected configuration was reused for MSL39 dataset without additional tuning. We trained for up to 200 epochs with batch size 32 using AdamW (Loshchilov and Hutter 2017) (learning rate $10^{-4}$, weight decay $10^{-4}$). Learning rates are scheduled with ReduceLROnPlateau (factor 0.1, patience 5, minimum lr $10^{-6}$), and early stopping is applied after 10 epochs without validation improvement. The total objective uses $\alpha_{\text{con}}=0.05$. For contrastive learning, we use temperature τ=0.1, domain dropout 0.1 to create two stochastic views, logsumexp aggregation, and negative weighting $\text{exp}(\lambda \delta_{ij})$ with λ=1.0. Figure S2 shows the hyperparameter sensitivity analysis curves. Distance-dependent margins are selected by rounding $\delta_{ij}$ into L+1 bins and mapping to a linear schedule in [0,1].

The D2T models we trained, along with the programs for training and testing, are available in a GitHub repository at https://github.com/mgtools/D2T. All the training and test datasets are also available in the D2T repository.

## 3 Results

### 3.1 Performance evaluation on double-stranded DNA viruses

We first report the results of the different models on datasets containing only double-stranded DNA viruses.

Table 1 shows the comparison of our methods (D2T and K+D2T) with ViTax and Kraken2 on the closed-set of double-stranded DNA viruses constructed from MSL38. The test-set is considered to be relatively easy as all the viruses in the test set belong to genera that have representatives in the training set. As the results show, all models achieved accurate predictions across the taxonomic ranks.

**Table 1:** Comparison of our methods (D2T and K+D2T) with ViTax and Kraken2 on the closed-set containing double-stranded DNA viruses.

Model	Rank	Acc.	Bal. Acc.	Prec.	Rec.	Macro F1	MCC
**ViTax**	Multi^a^	0.950					
	Genus^b^	0.892	0.852	0.863	0.852	0.855	0.896
**Kraken2**	Realm	0.973	0.692	0.797	0.692	0.739	0.899
	Kingdom	0.973	0.710	0.855	0.710	0.767	0.899
	Phylum	0.973	0.752	0.883	0.752	0.805	0.913
	Class	0.972	0.801	0.904	0.801	0.843	0.921
	Order	0.869	0.835	0.900	0.835	0.861	0.863
	Family	0.918	0.887	0.916	0.887	0.896	0.916
	Genus	0.935	0.933	0.928	0.930	0.927	0.935
**D2T**	Realm	0.992	0.784	0.756	0.784	0.768	0.969
	Kingdom	0.992	0.845	0.837	0.845	0.841	0.968
	Phylum	0.992	0.868	0.858	0.868	0.861	0.974
	Class	0.988	0.872	0.877	0.872	0.872	0.965
	Order	0.936	0.822	0.827	0.822	0.819	0.929
	Family	0.903	0.828	0.834	0.828	0.826	0.901
	Genus	0.807	0.732	0.673	0.730	0.689	0.806
**K+D2T**	Realm	0.996	0.793	0.773	0.793	0.783	0.983
	Kingdom	0.996	0.852	0.852	0.852	0.852	0.983
	Phylum	0.994	0.872	0.862	0.872	0.867	0.981
	Class	0.992	0.892	0.885	0.892	0.888	0.977
	Order	0.968	0.940	0.928	0.940	0.933	0.964
	Family	0.965	0.933	0.916	0.933	0.922	0.963
	Genus	0.951	0.942	0.930	0.939	0.930	0.951

a A sample was considered to be correctly classified if ViTax reported a label (any rank) that matches the ground-truth.

b Only samples that were classified at the genus level whose label matches the ground-truth were considered to be correct.

**Figure 4:**
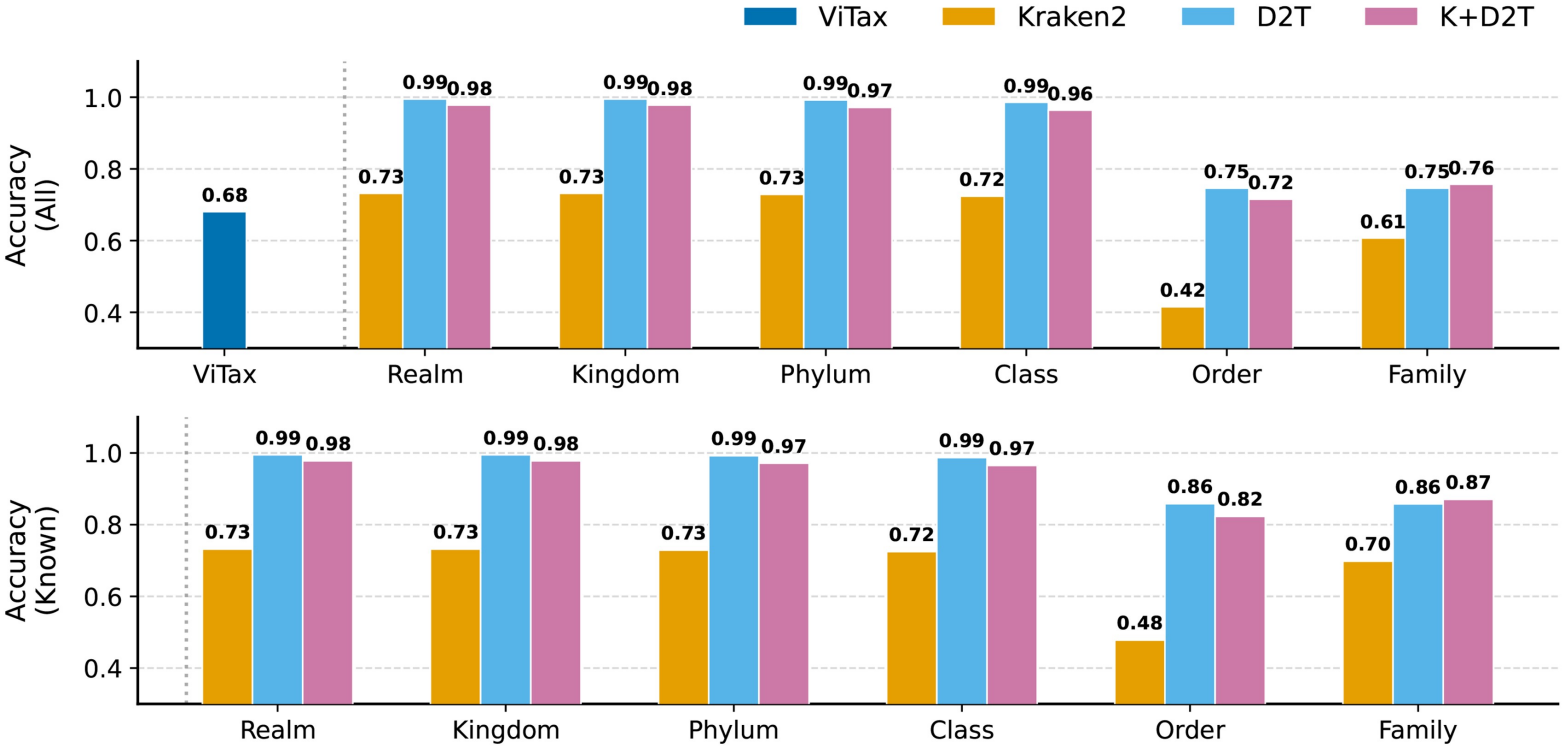
Performance comparison of the different models across different ranks of the viral taxonomy. Results for the MSL38 open-set cases are visualized here. ViTax produces a single prediction per sample at one taxonomic rank based on its confidence score so only one accuracy (across variable taxonomic ranks) is reported for this model. Genus-levels are omitted because the test set consists entirely of novel genera not seen during training, making genus-level classification inapplicable.

Table 2 and Fig. 4 summarize the performances of different models on the more challenging open-set cases. All models’ performance dropped. However, our method D2T retains its strong performance on these cases, especially at the higher taxonomic ranks with accuracy >0.98 at class level and up. At lower taxonomic level, D2T and K+D2T’s performances dropped but they still outperformed Kraken2 and ViTax with a large margin.

**Table 2:** Performance comparison on the MSL38 open-set in accuracy.

Rank	ViTax	**Kraken2**		**D2T**		**K+D2T**		Novel%
	Multi	All^a^	Known^b^	All	Known	All	Known	
Realm	0.681^c^	0.732	0.732	0.995	0.995	0.978	0.978	0.00%
Kingdom		0.732	0.732	0.995	0.995	0.978	0.978	0.00%
Phylum		0.729	0.729	0.992	0.992	0.972	0.972	0.00%
Class		0.724	0.725	0.986	0.987	0.964	0.965	0.13%
Order		0.415	0.478	0.746	0.858	0.715	0.823	13.08%
Family		0.607	0.698	0.746	0.858	0.757	0.871	13.02%

a Accuracy on all samples.

b Accuracy on known samples (known at the corresponding taxonomic rank, maybe unknown at lower ranks).

c Multi-level accuracy (the label reported by ViTax for a sample could be a high-level rank).

### 3.2 Performance evaluation on six realms of virus

We then tested the models on all viruses from different realms including DNA and RNA viruses to see if the performance of our methods hold when applied to diverse groups of viruses including six realms of DNA and RNA viruses. We constructed training and test datasets based on MSL39 and MSL40 for this task.

Table 3 shows the results on the MSL39 closed-set, where viruses in the test split share genera with those used for training. Compared with the results on double-stranded DNA viruses only (Table 1), Kraken2 exhibited a significant performance drop (e.g. realm-level accuracy decreased from 0.973 to 0.774), reflecting the increased difficulty of taxonomic assignment for non–double-stranded DNA viruses. In contrast, D2T remained robust, achieving near-perfect accuracy across all ranks (accuracy >0.95) except at the genus level (accuracy of 0.791). By leveraging the complementary strengths of D2T and Kraken2, K+D2T achieved a genus-level accuracy of 0.865 (Table 3).

**Table 3:** Comparison of our methods (D2T and K+D2T) with Kraken2 on the closed-set containing all types of viruses (MSL39 closed-set test).

Model	Rank	Acc.	Bal. Acc.	Prec.	Rec.	Macro F1	MCC
**Kraken2**	Realm	0.774	0.828	0.995	0.828	0.892	0.738
	Kingdom	0.775	0.812	0.998	0.812	0.882	0.741
	Phylum	0.772	0.721	0.990	0.721	0.803	0.763
	Class	0.774	0.628	0.913	0.612	0.697	0.768
	Order	0.684	0.633	0.897	0.624	0.697	0.709
	Family	0.735	0.585	0.720	0.529	0.573	0.756
	Genus	0.740	0.659	0.599	0.541	0.555	0.758
**D2T**	Realm	0.996	0.970	0.983	0.970	0.975	0.994
	Kingdom	0.996	0.993	0.961	0.993	0.974	0.994
	Phylum	0.990	0.935	0.984	0.935	0.940	0.988
	Class	0.987	0.835	0.868	0.813	0.822	0.985
	Order	0.974	0.828	0.872	0.817	0.828	0.973
	Family	0.958	0.760	0.701	0.688	0.683	0.950
	Genus	0.791	0.587	0.465	0.482	0.457	0.790
**K+D2T**	Realm	0.995	0.995	0.980	0.995	0.987	0.991
	Kingdom	0.995	0.994	0.971	0.994	0.982	0.992
	Phylum	0.988	0.936	0.981	0.936	0.945	0.985
	Class	0.985	0.875	0.922	0.852	0.866	0.983
	Order	0.972	0.867	0.909	0.855	0.867	0.971
	Family	0.959	0.828	0.772	0.750	0.748	0.958
	Genus	0.865	0.752	0.620	0.618	0.607	0.864

Table 4 summarizes results on more challenging settings: the open-set constructed from MSL39 and the new-set containing viruses newly collected in MSL40. Viruses in these datasets may be novel at different taxonomic ranks (see the novel% across ranks in Table 4). The results show a similar trend in which D2T retained near-perfect performance at the class rank and above (accuracy >0.98). Its performance declined at the order and family levels (with accuracies of 0.867 and 0.749, respectively) but still outperformed Kraken2 by a large margin (Kraken2 achieved accuracies of 0.192 and 0.321 at the order and family levels, respectively). Performance degradation on the new-set was more pronounced, which is expected given that a large fraction of viruses in the new-set are novel even at the order or family level. Nevertheless, D2T still achieved reasonable performance across ranks, particularly when considering the Accuracy (Known) metric.

**Table 4:** Performance evaluation on open-set and new-set datasets of viruses of all realms.

Rank	**Kraken2**		**D2T**		**K+D2T**		Novel%
	All^a^	Known^b^	All	Known	All	Known	
**Open-set based on MSL39, tested on model trained with MSL39**							
Realm	0.471	0.471	0.993	0.993	0.981	0.981	0.00%
Kingdom	0.474	0.474	0.995	0.995	0.984	0.984	0.00%
Phylum	0.474	0.474	0.991	0.991	0.978	0.978	0.00%
Class	0.470	0.471	0.982	0.984	0.966	0.969	0.22%
Order	0.192	0.197	0.867	0.892	0.850	0.874	2.74%
Family	0.321	0.353	0.749	0.826	0.735	0.810	9.26%
**New-set of viruses in MSL40, tested on model trained with MSL39**							
Realm	0.491	0.491	0.989	0.989	0.975	0.975	0.00%
Kingdom	0.490	0.494	0.981	0.989	0.966	0.973	0.78%
Phylum	0.509	0.539	0.914	0.967	0.899	0.952	5.55%
Class	0.489	0.540	0.871	0.962	0.861	0.952	9.50%
Order	0.073	0.162	0.385	0.858	0.378	0.843	54.16%
Family	0.064	0.174	0.300	0.816	0.293	0.796	63.23%

a Accuracy on all samples.

b Accuracy on known samples (known at the corresponding taxonomic rank, maybe unknown at lower ranks).

**Figure 5:**
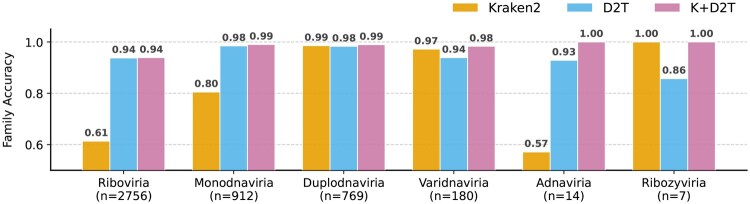
Performance breakdown across different viral realms. Shown here is the family-level accuracy for the MSL39 closed-set.

Figure 5 shows the breakdown of family-level accuracy across different viral realms. The comparison indicates that our methods (D2T and K+D2T) generally perform well across all realms. D2T’s performance on Ribozyviria is relatively weaker, likely due to the limited number of training samples available to build an accurate model for this group.

### 3.3 Using attention scores to identify taxonomically informative proteins

**Figure 6:**
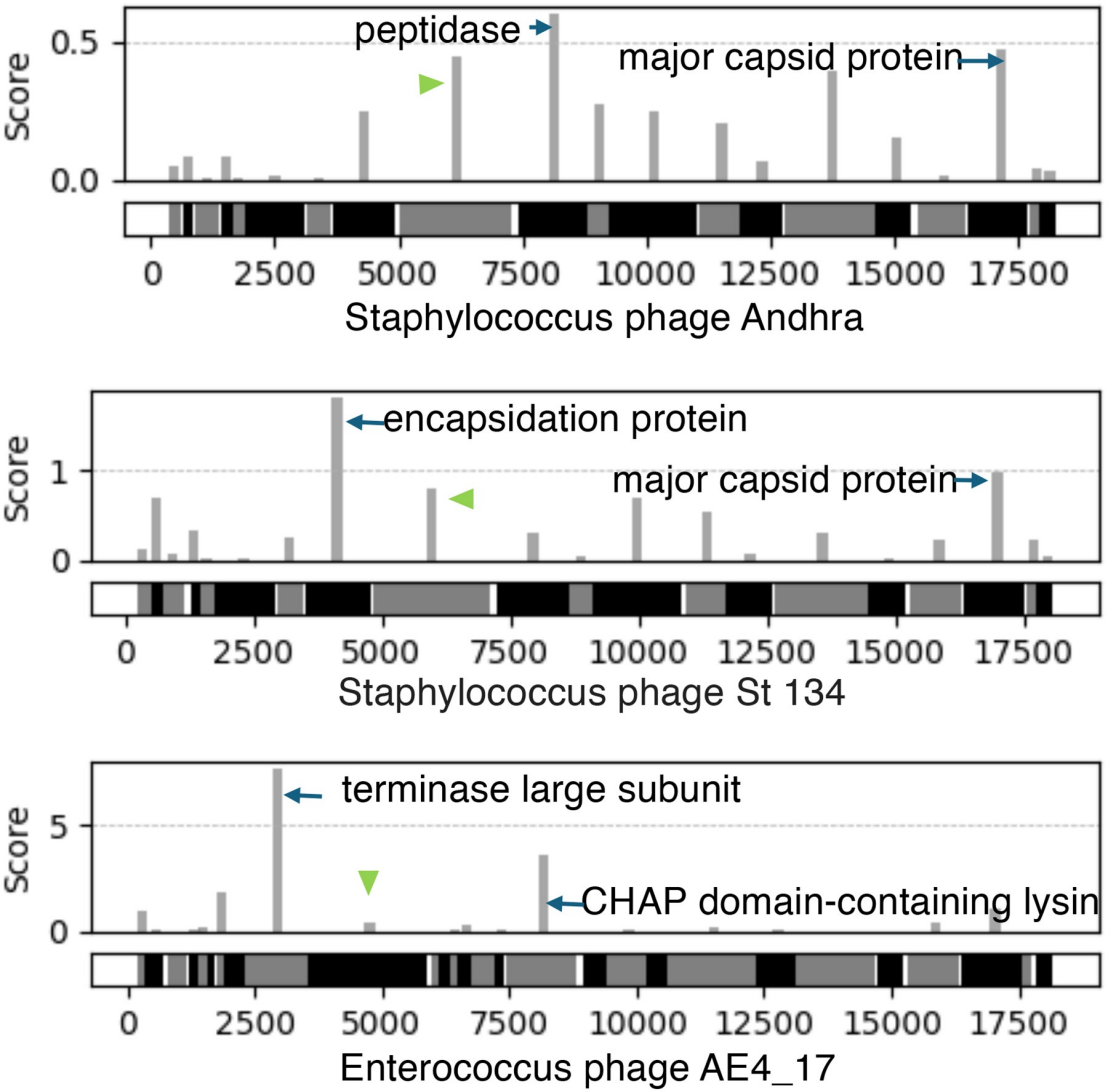
Examples illustrating D2T interpretability. Three representative examples are presented to demonstrate how D2T captures the differential contributions of individual proteins to taxonomic assignment. In each panel, viral genes are depicted as boxes with alternating black and gray colors along the bottom, with wider boxes representing longer genes, and x-tick labels marking their positions in the genome. The bar plot above shows the importance scores of the corresponding proteins. The two proteins with the highest contributions to the taxonomic assignment of each virus are highlighted with arrows along with their functions. Green triangles indicate DNA polymerases.

One potential advantage of using D2T for viral taxonomic classification is that we can utilize the attention scores to learn the differential contributions of proteins to taxonomic assignments. Figure 6 shows the taxonomic assignments of a few DNA viruses to illustrate the interpretability of D2T model.

The first example is *Staphylococcus* phage Andhra (isolate ID = 1002333) (Cater et al. 2017). Its genome is 18 546 bp in length and encodes 28 proteins, and it belongs to Duplodnaviria (realm), Heunggongvirae (kingdom), and Andhravirus (genus). The two proteins contributing most strongly to the taxonomic assignment of this virus are a peptidase (protein ID: AQT27832) and the major capsid protein (AQT27840).

The second example is *Staphylococcus* phage St 134 (Golosova et al. 2024). In this case, the encapsidation protein provides the strongest contribution to the taxonomic assignment, followed by the major capsid protein.

The third example is *Enterococcus* phage AE4_17, which infects antibiotic-resistant *E. faecalis* isolated from infected root canals (El-Telbany et al. 2021). The top contributing protein is the terminase large subunit, a key enzyme in many viruses that forms part of the DNA packaging machinery responsible for filling empty capsids with viral DNA.

Across all three examples, capsid proteins and terminases are among the most important proteins identified by D2T for taxonomic assignment. This observation is consistent with the literature, which indicates that virion morphogenesis modules provide suitable defining characters for DNA virus taxonomy (Simmonds et al. 2023). In contrast, DNA polymerases, despite being among the largest proteins encoded by these viruses, contribute less to taxonomic assignment. This is also consistent with previous findings showing that genes involved in genome replication are generally unsuitable for defining the realms of large DNA viruses, as even closely related viruses within the same realm often possess distinct replication modules.

### 3.4 Ablation study

We conducted an ablation study to examine the impacts of encoder architecture and the contrastive loss on the performance of D2T. To ensure a fair comparison, we applied the exact same hyperparameters (e.g. learning rate, batch size) to all ablated models. We compared MLP encoder with the Transformer encoder under the same configuration utilizing contrastive loss. We also compared the Transformer encoder trained with or without contrastive loss. Table 5 summarizes the results on the MSL38 closed-set. The results show that the Transformer encoder outperforms the MLP encoder across all ranks, and the inclusion of contrastive loss proved to be effective. The Transformer encoder trained with contrastive loss demonstrated better performance across all metrics compared to the same encoder without it. These results demonstrate that the combination of a Transformer encoder and contrastive loss yields the most robust representations for taxonomy classification.

**Table 5:** Ablation study comparing encoder architecture and contrastive loss (MSL38 closed-set).

Rank	**Accuracy**			**Balanced Accuracy**			**Macro F1**		
	MLP+CL	**T** − **CL**	**T** + **CL**	**MLP** + **CL**	**T** − **CL**	**T** + **CL**	**MLP** + **CL**	**T** − **CL**	**T** + **CL**
Realm	0.982	0.987	0.992	0.693	0.731	0.784	0.732	0.744	0.778
Kingdom	0.980	0.987	0.992	0.716	0.659	0.845	0.767	0.692	0.841
Phylum	0.979	0.987	0.992	0.749	0.746	0.868	0.788	0.767	0.861
Class	0.976	0.984	0.988	0.698	0.703	0.872	0.736	0.726	0.872
Order	0.883	0.929	0.936	0.600	0.806	0.822	0.604	0.796	0.819
Family	0.798	0.884	0.903	0.587	0.700	0.828	0.602	0.693	0.826
Genus	0.681	0.732	0.807	0.528	0.586	0.729	0.498	0.544	0.688

T: Transformer encoder; MLP: multi-layer perceptron encoder; CL: contrastive loss.

+CL indicates that contrastive loss is used; –CL indicates that contrastive loss is not used.

In addition, we tested if skani, a recently developed tool for fast ANI calculation, can be utilized for taxonomic assignment at genus level. We adopted a nearest-neighbor strategy to use the skani search results (using -n 1 option) to identify the single best-matching reference (top-1) based on ANI, and then assign its taxonomy to the query. It is important to note that skani employs stringent internal filters, discarding matches with an aligned fraction below 15% and ANI below 80%. Queries failing to meet these thresholds yielded no results and were counted as misclassifications. Evaluation using MSL38 closed-set showed that skani-based ANI didn’t yield good results: at the genus level, skani achieved accuracy of 0.774, a significant drop compared to 0.935 achieved by Kraken2 on the MSL38 closed-set. As a result, we only compared our performance with Kraken2 above, and developed our hybrid approach using Kraken2.

## 4 Discussion

By leveraging domain fingerprints, our D2T model achieved accurate taxonomic assignments for viruses across taxonomic ranks and across different viral groups (both DNA and RNA viruses). D2T performs particularly well at higher taxonomic ranks, where methods relying on genomic information, either sequence similarity (e.g. Kraken2) or DNA language models (e.g. ViTax), tend to underperform. Despite its strong predictive accuracy, there remains room for improvement. In some test cases, especially in open-set settings, balanced accuracy and F1 score remain relatively low, likely due to class imbalance. Strategies such as oversampling rare classes and undersampling dominant classes may further improve performance. In addition, we plan to develop confidence scores to explicitly flag out-of-distribution inputs and better distinguish novel viral groups.

Here, we adopted a simple strategy to leverage the complementary strengths of Kraken2 and D2T to achieve accurate taxonomic classification across all ranks. Lower ranks (e.g. genus) benefit from sequence similarity–based methods such as Kraken2, whereas higher ranks benefit from the use of protein contextual information captured by D2T.

Examination of misclassified cases suggests that domain scarcity may have contributed to high-confidence errors across all ranks (see Tables S1 and S2). Meanwhile, Genus-level errors often persist even with substantial domain counts. This suggests that domain fingerprints may be effective at capturing broad evolutionary signals but may lack the fine-grained sequence specificity required for genus-level discrimination. This divergence reinforces the necessity of our hybrid K+D2T approach.

It may be worthwhile to explore additional sequence similarity–based approaches, including the use of the skani tool, to develop more accurate hybrid methods. As skani was primarily designed for fast ANI calculation, our current nearest-neighbor strategy may not fully exploit its capabilities. Another promising direction is to integrate domain-level features that are informative for higher-level taxonomic assignment with fine-grained genomic (or protein) sequence features that are critical for genus-level classification into a unified model for viral taxonomy across all ranks.

## Supplementary Material

btag291_Supplementary_Data

## Data Availability

All training and test datasets are available in D2T github repository at https://github.com/mgtools/D2T.
