# Supplementary material for: A novel transformer model of protein domains for viral taxonomy classification
Source: Bioinformatics. 2026 Jul 7;42(Suppl 1):btag291. doi: 10.1093/bioinformatics/btag291 (PMC13341119; doi:10.1093/bioinformatics/btag291)
Supplement: btag291_Supplementary_Data [file btag291_supplementary_data.pdf]

Supplementary Material

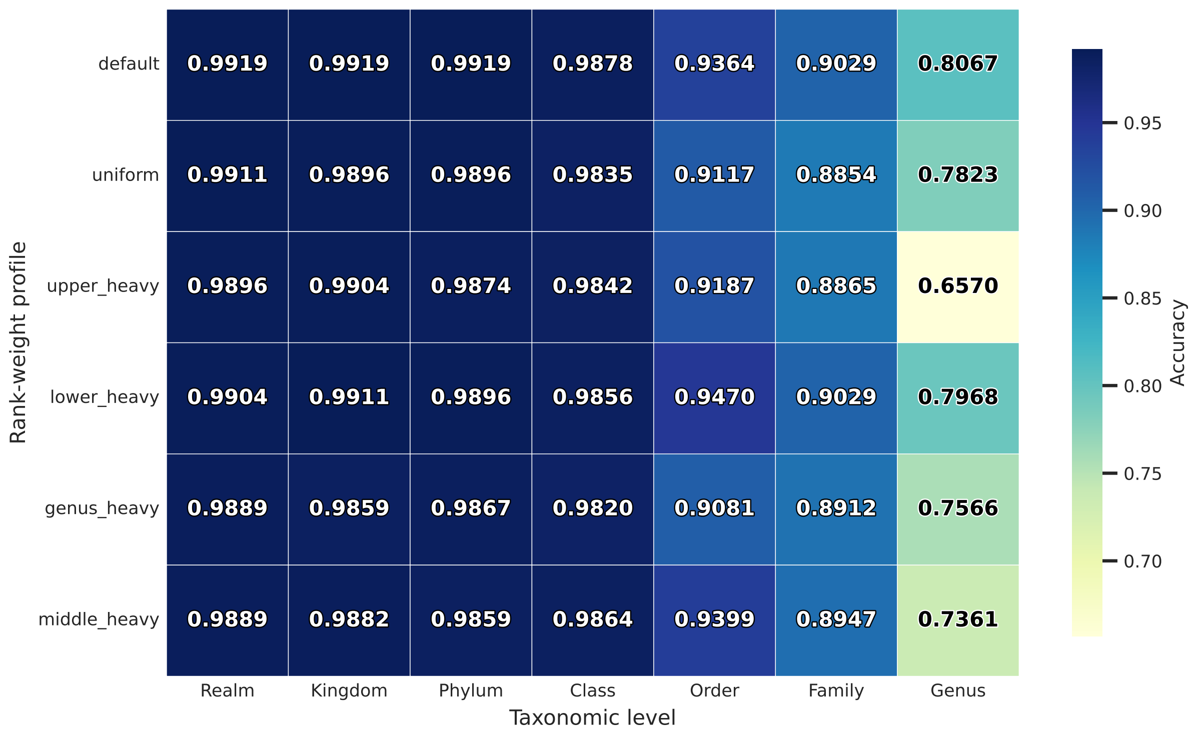

Figure S1: Rank-weight sensitivity on MSL38, evaluated in a one-factor-at-a-time manner with all other settings fixed. The weights indicate the relative contribution of each taxonomic rank to the total classification loss. We tested six hierarchical loss-weight profiles across Realm to Genus and reported Accuracy at each taxonomic level as heatmap. The profiles are: default [0.18, 0.17, 0.15, 0.13, 0.11, 0.10, 0.16], uniform [1/7, . . . , 1/7], upper_heavy [0.22, 0.20, 0.16, 0.14, 0.12, 0.09, 0.07], lower_heavy [0.07, 0.09, 0.12, 0.14, 0.16, 0.20, 0.22], genus_heavy [0.03, 0.05, 0.08, 0.12, 0.17, 0.20, 0.35], and middle_heavy [0.10, 0.12, 0.16, 0.24, 0.16, 0.12, 0.10].

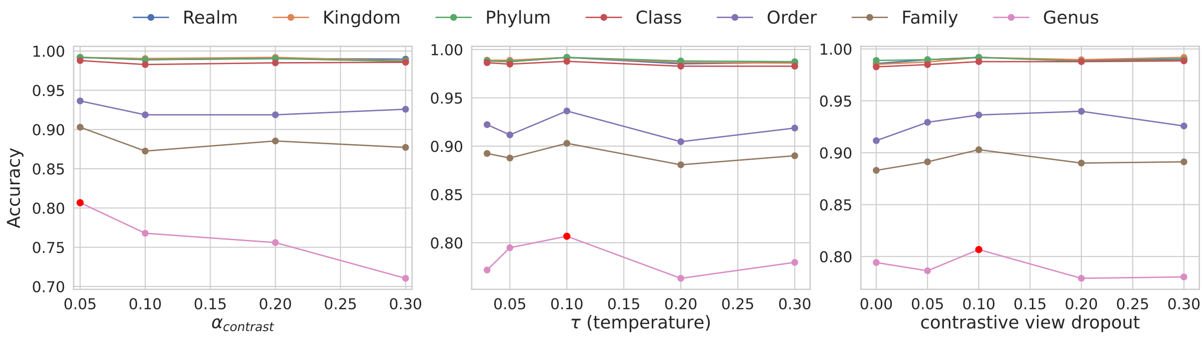

Figure S2: Sensitivity curves on MSL38 dataset for α_{contrast}, τ (temperature), and dropout rate for contrastive view, evaluated in a one-factor-at-a-time manner with all other settings fixed. Each line shows Accuracy at a taxonomic level (Realm to Genus), and the red dot indicates the best Genus-level performance.

Table S1: Summary of accuracy and prediction confidence across taxonomic levels on the MSL39 closed-set. The prediction confidence is defined as the predicted class probability (softmax top-1 score). The table shows the total number of samples (n), accuracy, number of misclassified samples (Wrong n), mean confidence for correct and wrong predictions, and the count and rate of high-confidence errors (confidence ≥ 0.90).

**Level**	**Total (n)**	**Accuracy**	**Wrong (n)**	**Mean Conf (Correct)**	**Mean Conf (Wrong)**	**High-Conf Wrong (n)**	**High-Conf Wrong Rate**
Realm	5496	0.9960	22	0.9996	0.9115	15	0.6818
Kingdom	5476	0.9962	21	0.9995	0.8104	10	0.4762
Phylum	5469	0.9901	54	0.9988	0.7752	21	0.3889
Class	5517	0.9871	71	0.9981	0.7248	26	0.3662
Order	3970	0.9741	103	0.9945	0.7149	36	0.3495
Family	4870	0.9517	235	0.9833	0.6027	39	0.1660
Genus	5726	0.7911	1196	0.8938	0.4502	90	0.0753

Table S2: Failure case analysis by taxonomic level and confidence group on the MSL39 closed-set. Comparison of domain counts (mean, Q1, median, Q3) among viruses classified by taxonomic rank and prediction confidence.

**Taxonomic Rank**	**Group**	**n**	**Mean**	**Q1**	**Median**	**Q3**
Realm	Correct [a]	5474	107.91	15.00	35.0	142.00
	Wrong (low-conf) [b]	7	22.00	4.00	6.0	11.00
	Wrong (high-conf) [c]	15	4.87	1.00	3.0	5.50
Kingdom	Correct	5455	108.25	15.00	36.0	142.00
	Wrong (low-conf)	11	15.00	1.00	6.0	7.50
	Wrong (high-conf)	10	7.40	3.00	4.0	4.75
Phylum	Correct	5415	108.88	15.00	36.0	143.00
	Wrong (low-conf)	33	17.15	3.00	6.0	17.00
	Wrong (high-conf)	21	20.05	5.00	15.0	19.00
Class	Correct	5446	113.17	15.00	37.0	146.00
	Wrong (low-conf)	45	63.49	4.00	10.0	27.00
	Wrong (high-conf)	26	23.27	5.25	16.5	29.25
Order	Correct	3867	56.57	14.00	22.0	41.00
	Wrong (low-conf)	67	71.88	6.00	17.0	42.00
	Wrong (high-conf)	36	20.67	4.00	17.5	33.00
Family	Correct	4635	90.90	14.00	26.0	59.00
	Wrong (low-conf)	196	62.89	11.75	26.0	39.00
	Wrong (high-conf)	39	25.21	4.00	14.0	27.50
Genus	Correct	4530	115.96	15.00	37.0	151.00
	Wrong (low-conf)	1106	82.42	14.00	27.0	57.75
	Wrong (high-conf)	90	59.12	7.00	14.5	24.00

[a] Correctly classified samples.
[b] Misclassified samples with confidence < 0.90.
[c] Misclassified samples with confidence ≥ 0.90.
